# Preoperative SPECT/CT + intraoperative CT fusion enabling surgical augmented reality to target sentinel lymph node in endometrial cancer

**DOI:** 10.1186/s40658-022-00506-7

**Published:** 2022-11-22

**Authors:** Lise Lecointre, Juan Verde, Fabrice Hubele, Julien Salvadori, Laurent Goffin, Chérif Akladios, Benoît Gallix

**Affiliations:** 1 Department of Gynecologic Surgery, University Hospitals of Strasbourg, Avenue Molière, 67200 Strasbourg, France; 2 Institute of Image-Guided Surgery, IHU-Strasbourg (Institut Hospitalo-Universitaire), Strasbourg, France; 3 ICube UMR 7357 - Laboratoire Des Sciences de l’ingénieur, de l’informatique et de l’imagerie, CNRS, Université de Strasbourg, Strasbourg, France; 4 Nuclear Medicine and Molecular Imaging, Institut de Cancérologie de Strasbourg Europe (ICANS), University Hospitals of Strasbourg, Strasbourg University, 67200 Strasbourg, France; 5 Department of Diagnostic Radiology, McGill University, Montreal, Canada

## Abstract

**Purpose:**

To establish a proof-of-concept study using a phantom model to allow the fusion of preoperative single-photon emission computed tomography (SPECT) combined with computed tomography (CT), also known as SPECT/CT, with intraoperative CT, enabling the application of an augmented reality (AR) surgical guidance system for pelvic sentinel lymph node (SLN) detection in endometrial cancer patients.

**Methods:**

A three-dimensional (3D) pelvic phantom model printed in a gelatin-based scaffold including a radiopaque pelvis, a vascular tree mimicking the iliac vessels, two 3D-printed fillable spheres representing the target pelvic sentinel lymph nodes, and a calibration board was developed. A planar with SPECT/CT lymphoscintigraphy and CT were performed independently on the model. We performed all the necessary steps to achieve the fusion between SPECT/CT and CT. Then, we performed a laparoscopy of the pelvic anatomy on the phantom model to assess in real time the overlay of the recording on the anatomical structures and AR guidance system performance.

**Results:**

We have successfully completed all the steps needed to fuse the two imaging procedures. This allowed us to apply, in real time, our surgical guidance system with the coverage rate of the visible surface by the augmented reality surface, respectively, on the left SLN 99.48% and on the right SLN 99.42%.

**Conclusion:**

Co-registration and real-time fusion between a preoperative SPECT/CT and intraoperative CT are feasible. The metric performance of our guidance system is excellent in relation to possible SPECT/CT and CT fusion. Based on our results, we are able to translate the technology to patients, and we initiated a clinical study to evaluate the accuracy of the AR guidance system for endometrial cancer surgery, with a correlation with indocyanine green (ICG)-based technique, representing the gold standard today in the intraoperative detection of SLN in endometrial cancers, despite various limitations.

## Background

The assessment for metastases of the pelvic lymph nodes (LN) is critical for oncological management patients, as the metastatic status of LN determines the stage and prognosis. The introduction of the concept of sentinel lymph node (SLN) has drastically changed the management of these patients. The SLN is the first node (or nodes) on the draining pathway from the primary site, thus the most likely location to be metastatic. Regarding endometrial cancer (EC) staging, SLN is recommended by the last edition of the European Guidelines [1]. However, SLN procedures for EC have several limitations, most importantly its reliability, accuracy, and the potential procedure-related complications [2–6].

Recent studies reported an increased detection rate of SLN using indocyanine green (ICG) intraoperatively [2]. Although some studies brought up high SLN detection rates using this method, other authors reported problems, being one of the biggest the detection of *multiple* SLN (5 or more). This makes very difficult to determine the *true* SLN, thus decreasing accuracy when compared to preoperative techniques using radioisotopes which usually retrieve a single SLN [7]. Even further, the dissection of multiple LN may lead to undesired complications as lymphedema, and deep-seated SLNs, or SLN in obese patients may be overlooked using ICG favoring recurrences and tumoral persistence.

Preoperative medical images bring up useful information regarding SLN location, their relation key anatomical structures, and even functional information; thus, they are useful for surgeons to plan the procedure. Among these methods, single-photon emission computed tomography (SPECT) combined with computed tomography (CT), also known as SPECT/CT, is well known due to its high detection rate, ranging from 76 to 90% [7–10].

The functional aspects of SPECT imaging enhance sensitivity [10], while anatomical information comes from CT, allowing the spatial location of the findings. Additionally, other technique using hybrid tracers (ICG-[^99m^Tc] Tc-albumin) by transvaginal ultrasound-guided myometrial injection of radiotracer (TUMIR) was developed to boost the benefits of the radiotracer and the fluorescence methods with a single tracer [11], enabling SLN mapping preoperatively.

Nowadays, it is possible to augment surgical video streams in real time by overlying preoperative ancillary data, like medical images [12]. Augmented reality (AR) systems overlying 2D/3D patient-specific models to laparoscopic video images in real time have already shown their value in aiding surgeons to perform more precise and safe dissections.

However, due to surgical changes and dynamics as motion and deformation, the translation of preoperative SPECT/CT information into the intraoperative settings remains an open problem.

In this study, we present a proof-of-concept study using a phantom model to enable the fusion of preoperative SPECT/CT with intraoperative CT, enabling accurate augmentations.

## Phantom model

A CT scan from a female patient was post-processed to create a model capable of being build using additive manufacturing technologies (a.k.a., 3D printing). A printable mesh was created using semiautomatic segmentations with manual corrections (3D slicer—[13]). Multiple post-processing steps to improve multimodal imaging compatibility were performed, such as radiopacity, smoothing edges, and mounting. A synthetic vascular tree mimicking iliac vessels was created using tubing, filled with contrast agents (iodinated contrast + blue dye), and finally mounted. Two 3D-printed spheres were positioned at the end to simulate SLNs, using different sizes (8 mm left–15 mm right, respectively), enabling the posterior injection of radioactive isotopes.

**Fig. 1:**
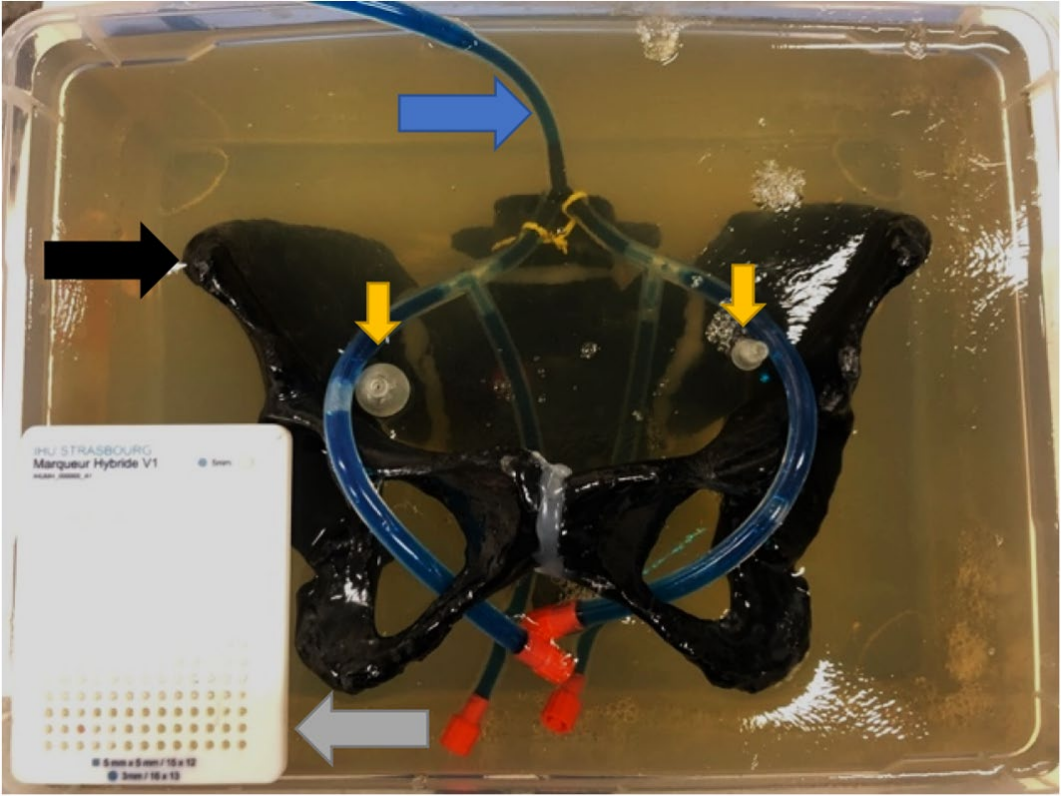
Phantom model including a 3D-printed pelvis (black), a vascular tree mimicked using standard tubing (blue), two spheres simulating target SLN (yellow), and a calibration device (gray) amalgamated by a gelatin-based support. SLN, sentinel lymph node

The system was conceived as mechanically stable, simulating the retroperitoneal environment, using a gelatin-based scaffold (pig gelatin, manufacturer Louis François). Finally, a calibration device was integrated to compute the transformations across different medical imaging methods (Fig. 1).

### Preoperative SPECT/CT

**Fig. 2:**
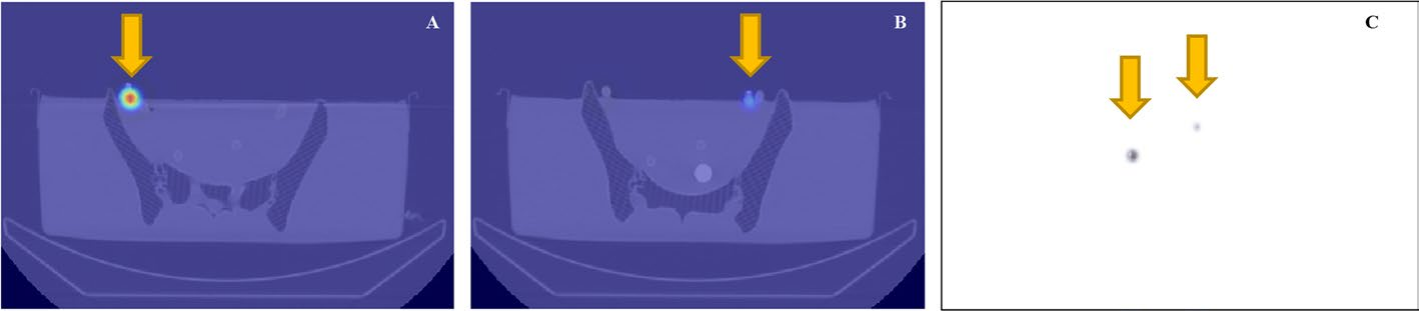
SPECT/CT and planar lymphoscintigraphy images acquired from the phantom model. **A** Axial fused SPECT/CT images showing a SLN on the right, **B** same on the left, **C** planar lymphoscintigraphy including both SLNs. SPECT/CT, single-photon emission computed tomography combined with computed tomography; SLN, sentinel lymph node

In the imaging facility (Nuclear Medicine and Molecular Imaging Department at the Strasbourg University Hospital), each sphere was filled with 12 MBq/mL of a homogeneous ^99m^Tc solution. SPECT/CT acquisition was performed with a GE Discovery NM CT 870 DR, equipped with LEHR (low-energy high-resolution) collimators and 5/8″-thick crystal. Sixty projections of 30 s per projection were acquired with a 128 × 128 matrix size (pixel of 4.4 mm). A photopeak energy window of 20% width centered on 140.5 keV and a scatter energy window of 10% width centered on 120 keV. Images were reconstructed with OSEM (ordered subset expectation maximization) algorithm (8 iterations and 8 subsets) with all corrections available (resolution recovery, attenuation, and scatter). Attenuation correction was performed with the CT images, and scatter correction was performed with the dual energy window method. The results are shown in Fig. 2. The SPECT/CT images were used to provide a visual assessment of the SLN’s location to make an indicative reference and not intended to perform any quantification.

### Intraoperative CT

**Fig. 3:**
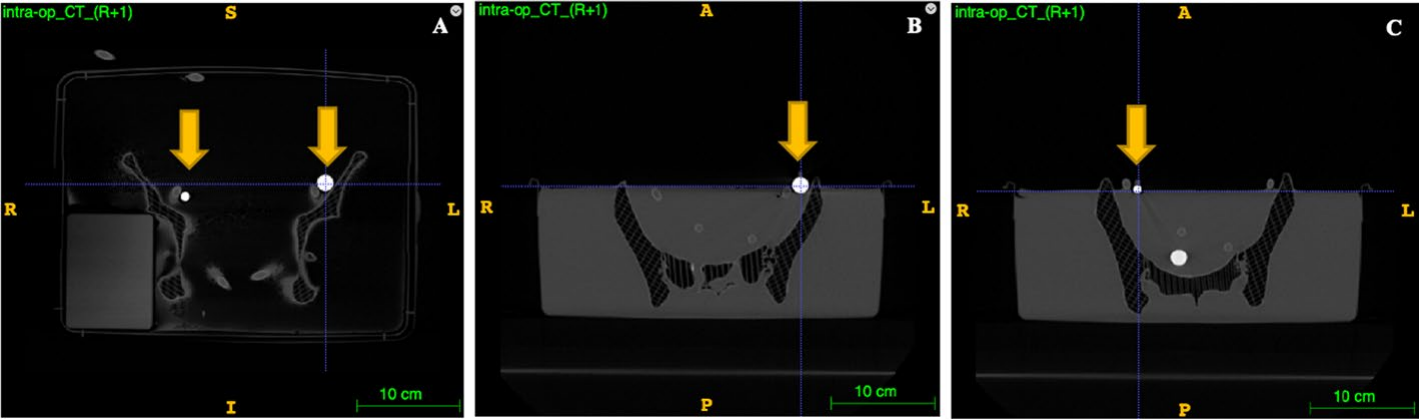
Computed tomography of the model. Coronal (**A**) and axial CT images showing the target SLNs in both sides (arrows: right SLN (**B**) and left SLN (**C**)) in arterial phase. CT, computed tomography; SLN, sentinel lymph node

Due to the different characteristics and specifications of our phantom model when compared to human’s, a standard low-dose and non-contrasted CT scan was sufficient to complete the entire pipeline, including initial segmentation steps and subsequent transformations [13, 14]. Also, a new set of printed spheres simulating SLNs were filled with iodinated contrast medium to improve visibility and downstream tasks (Fig. 3).

## Image registration

**Fig. 4:**
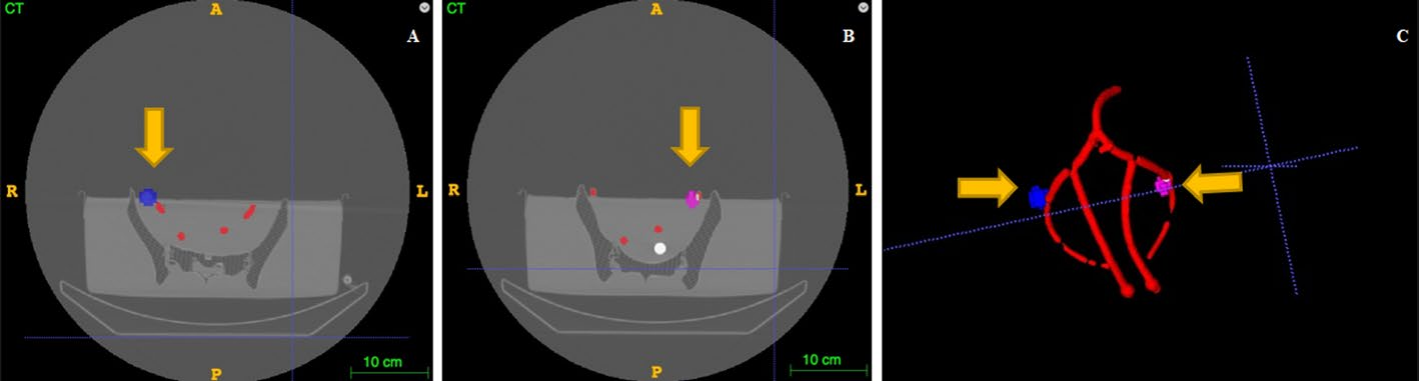
Registration of SPECT/CT and CT images using itk-SNAP® 3.6.0. The fusion CT image with the right (**A**) and left (**B**) SLN after loading of the SPECT/CT and after the synchronization of the CT with the SPECT/CT, achieved by the image fine-tuning over the pelvis. 3D reconstruction of vascular tree and both pelvic nodes in the same reference area (**C**). SPECT/CT, single-photon emission computed tomography combined with computed tomography; CT, computed tomography; SLN, sentinel lymph node

Several steps were necessary to achieve a consistent fusion of the initial SPECT/CT and the CT. First, we started with the segmentation of all target structures, including the two SLNs on the CT scan. Then, an automatic registration with an open-source software (itk-SNAP® 3.6.0—stable version) (after an initial manual alignment) using the mutual information of the two images (CT/SPECT) was performed. After a visual validation relying on bone structures, a 4 × 4 transformation matrix was extracted and used to achieve the linear interpolation between the two images and segmented structures. Finally, the segmented SPECT structures (SLN) were brought to the same images space using the same matrix (Fig. 4).

**Fig. 5:**
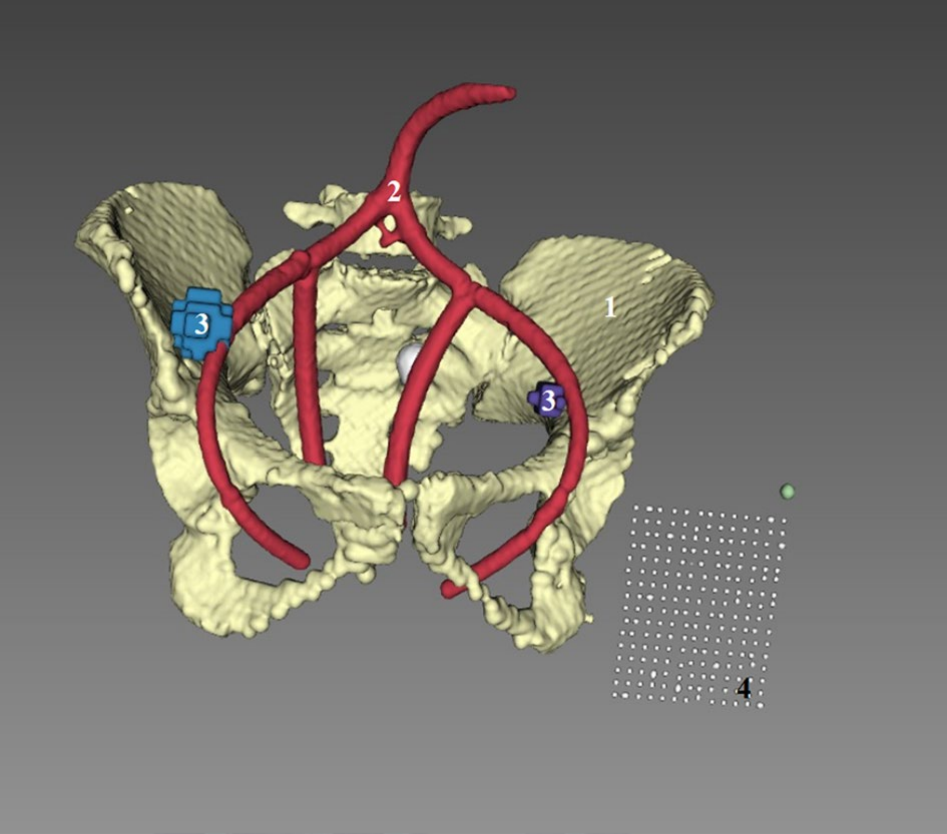
Results of the segmentation process, after registration. Segmentation of CT images with 3D reconstruction of pelvic structures (pelvic bones (1), arteries (2), lymph nodes (3), and calibration device (4). CT, computed tomography

Manual segmentations of relevant anatomical landmarks, performed by a trained surgeon, were provided for both images. Subsequently, the CT images were post-processed to create accurate 3D segmentations enabling volumetric computations and the creation of meshes to be superimposed as augmentations. The open-source software (itk-SNAP® 3.6.0—stable version and 3D Slicer® (version 4.13.0)) were used to create these segmentations via an automatic approach which was then corrected manually if needed (Fig. 5).

### Augmented reality application

**Fig. 6:**
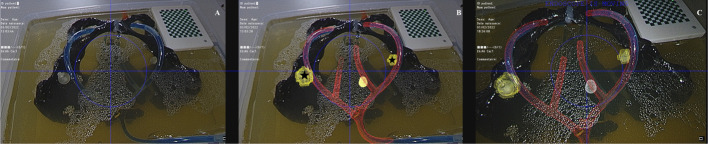
Registration of SPECT/CT and CT. Laparoscopic view of the pelvic anatomy on phantom model (**A**) enhanced by real-time AR overlay of SLNs and anatomical landmarks, with SLNs marked with a black asterisk on the left and right (**B**) and in higher magnification to assess the overlay of the registration on anatomical structures and its performance (**C**). SPECT/CT, single-photon emission computed tomography combined with computed tomography; CT, computed tomography; SLN, sentinel lymph node

Finally, the surgical simulation of the procedure consisted in the laparoscopic identification on the phantom of the SLNs marked, and the surrounding pelvic lymphadenectomy landmarks including the external and internal iliac arteries. Accuracy of registration was compared between the standard (Fig. 6A) and the AR assistance approaches (Fig. 6B, C).

**Fig. 7:**
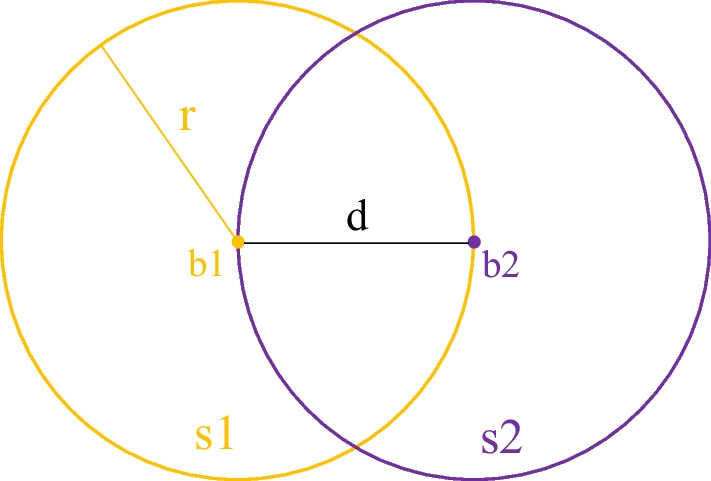
Graphical representation of the normalized barycenter distance method. *b*1 barycenter of the surface *s*1 of the simulated sentinel lymph node visualized by augmented reality, *b*2 barycenter of the surface *s*2 of the simulated sentinel lymph node visualized in the endoscopic image, *d* the distance between the two barycenters *b*1 and *b*2 and *r* the theoretical radius of the surface *s*1

On the endoscopic image, we measured in pixels^2^ the surface *s*1 corresponding to the lymph node visualized by augmented reality and the surface *s*2 corresponding to the lymph node in the endoscopic image, as well as the barycenter of each surface, respectively, *b*1 and *b*2, whose coordinates are expressed in pixels. Barycenters correspond to 2D coordinates, expressed in pixels unities. From the pseudo-radius of each surface, expressed in pixels (theoretical or average radius for any surface), *r* = √ (surface/Pi), we compute the ratio of barycenter’s distance (*b*1 and *b*2) with the pseudo-radius (*r*): Distance (*b*1, *b*2)/*r*. The smaller the ratio (close to 0), the more centered the registration is on the lymph node (Fig. 7).

This ratio is 0.2 on the left and 0.11 on the right, which means that the two augmented reality barycenters are included in visible surfaces (the ratio is less than 1) and that barycenters are, respectively, 0.2 times (1/5th) and 0.11 times (1/9th) of the pseudo-radius of each target center. We also compute the coverage rate of the visible surface by the augmented reality surface, respectively, on the left 99.48% and on the right 99.42%, as follows: *s*1 ∩ *s*2/*s*2.

### Clinical perspectives

Based on our results, we started the clinical translation process, and we initiated a clinical study aiming at evaluating the accuracy of the AR guidance system for endometrial cancer surgery, with a correlation to standard ICG-based technique. As a de-risking strategy and initial step, we considered potential drawbacks related to surgical dynamics (e.g., motion, deformation, etc.), impacting the precision under unforeseen surgical constraints. Therefore, we have suggested the use of the proposed solution intraoperatively in parallel on a side screen, hence not available for the surgical team initially.

The project will use bone structures for the registration between the preoperative SPECT/CT and the intraoperative CT as described above. The segmentation of pre-operative images will be performed semiautomatically under, and manually corrected on demand by a trained surgeon. The same algorithms for robotic/endoscope registration will be used, and SPECT/CT segmentations (i.e., SLN, vascular structures, ureter, uterus, and bones) will be superimposed in the endoscopic video stream in real time.

## Data Availability

The datasets generated and/or analyzed during the current study are available from the corresponding author in the ARGyS repository.
